# Introduction of flexometallic cuffed endotracheal tube through COBRA perilaryngeal airway

**DOI:** 10.4103/0019-5049.76585

**Published:** 2011

**Authors:** Lulu Fatema Vali, Sonali Khobragade

**Affiliations:** Department of Anaesthesiology, Superspeciality and Government Medical College and Hospital, Nagpur, Maharashtra, India

Sir,

A 45-year-old male patient weighing 84 kg with diagnosis of pituitary macroadenoma posted for sublabial rhinoseptal trans-sphenoidal pituitary macroadenoma excision. Patient was having features of acromegaly with heavy jaw and large tongue. Airway assessment revealed adequate mouth opening with mallampatti scale class III. Patient was pre-oxygenated for 5 minutes. He was premedicated with intravenous (IV) pantoprazole 40 mg, IV glycopyrrolate 0.2 mg, IV dexamethasone 8 mg, IV midazolam 1 mg and IV fentanyl 50 mcg. The patient was induced with IV propofol 2 mg/kg and IV rocuronium bromide 0.6 mg/kg. Laryngoscopy was performed but we could not intubate the patient as it was Cormark-Lehane grade IV on direct laryngoscopy. We inserted the COBRA perilaryngeal airway (PLA) no. 4 with which patient was successfully ventilated and it was confirmed on auscultation. We were not successful in passing flexometallic tube through COBRA PLA. Hence, we passed a lubricated bougie through COBRA PLA into trachea. After removing COBRA PLA, we did larygoscopy and threaded the cuffed flexometallic tube no. 8.0 over bougie. Bougie was removed and flexometallic endotracheal tube position was confirmed with auscultation and capnography.

**Figure 1:**
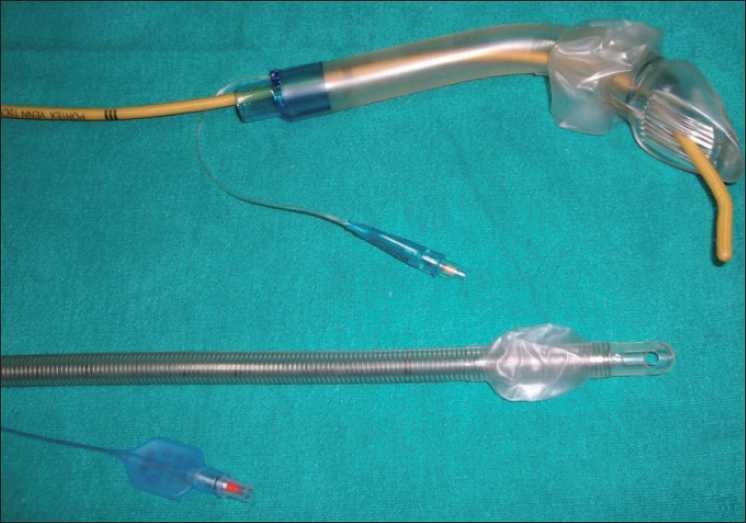
COBRA PLA with bougie

In a study conducted by Lee and others,[1] after inserting COBRA PLA, fibre-optic bronchoscope was passed through it and then blind as well as fibre-optic guided intubation was done; but passing a bougie through COBRA PLA [Figure 1] and threading a flexometallic tube over bougie with resultant successful intubation in difficult airway management was a unique experience to be shared.
